# 免疫检查点抑制剂相关肾脏不良反应的临床诊治建议

**DOI:** 10.3779/j.issn.1009-3419.2019.10.07

**Published:** 2019-10-20

**Authors:** 维 邱, 可 郑, 汉萍 王, 晓燕 斯, 晓彤 张, 雪梅 李, 力 张

**Affiliations:** 1 100730，北京中国医学科学院，北京协和医学院北京协和医院肾内科 Department of Nephrology, Peking Union Medical College Hospital, Chinese Academy of Medical Sciences, Beijing 100730, China; 2 100730，北京中国医学科学院，北京协和医学院北京协和医院呼吸内科 Department of Respirology, Peking Union Medical College Hospital, Chinese Academy of Medical Sciences, Beijing 100730, China

**Keywords:** 免疫检查点抑制剂, 免疫相关肾毒性, 急性肾小管间质性肾炎, Immune checkpoint inhibitors, Immune-related nephrotoxicity, Acute tubulointerstitial nephritis

## Abstract

免疫检查点抑制剂（immuno-checkpoint inhibitors, ICIs）正越来越多地应用于临床肿瘤治疗，显著改善了患者预后。T细胞过度活化及相关信号通路的激活，可能引起药物相关的免疫相关不良反应（immune-related adverse effects, irAEs）。其中肾脏免疫相关不良反应相对罕见，但也存在严重甚至致命的副作用。本文分析ICIs免疫相关肾损伤的发病率、临床表现及肾脏病理表现，着重讨论诊断和治疗原则。因存在诸多继发因素须与ICIs免疫相关肾损伤相鉴别，必要时应行肾活检以决定重要药物的治疗决策。

## Abstract (en)

Immune checkpoint inhibitors (ICIs) have been used more and more Increasingly in clinical oncology treatment, which has significantly improved the prognosis of cancer patients. Over-activation of T cells and related signaling pathways may cause immune-related adverse effects. Renal immune side-effects are relatively rare, but some of them are serious and fatal. This review analyses of the Incidence, clinical and pathological manifestations of ICIs-induced renal injury, and focuses on the differential diagnosis and treatment. Because there are many secondary factors that need to be differentiated from immune mechanism, renal biopsy should be performed if necessary to determine the important decision.

近年来，免疫检查点抑制剂（immuno-checkpoint inhibitors, ICIs）的研究有力推动了肿瘤治疗进展。ICIs的重要机制为阻断T细胞表面的细胞毒T淋巴细胞相关抗原4（cytotoxic T lymphocyte-associated antigen-4, CTLA-4）（代表药物ipilimumab）或程序化细胞死亡蛋白（programmed death 1, PD-1）/程序化细胞死亡配体-1（programmed death ligand 1, PD-L1）（代表药物nivolumab与pembrolizumab）信号通路，从而恢复其杀伤肿瘤细胞的活性^[1]^。然而过度的T细胞激活，可能导致免疫相关不良反应，从而引起正常组织损伤。既往认为肾脏副作用虽相对罕见，仍可能存在严重甚至致命的不良反应^[2]^。本文探讨ICIs免疫相关肾毒性的临床病理特点，联合多种辅助检查手段提高诊断效率，提出合理的治疗方案从而改善ICIs免疫相关肾脏不良反应的预后，为将来ICIs更广泛更安全地应用于抗肿瘤治疗提供参考。

## 1 发病率

相关统计表明ICIs肾损伤的总体发生率约为2.2%，其中3级或4级急性肾损伤（定义为血清肌酐>基线3倍，增加至>4 mg/dL或需要肾脏替代治疗）的发病率为0.6%。nivolumab治疗中肾脏不良反应发生率约1.9%，pembrolizumab约1.4%，而ipilimumab治疗中约2.0%^[3]^。增加剂量^[4]^及联合用药^[5, 6]^均可能带来更严重的肾毒性。更完善的随访及监测在客观上提高了诊断率。新近研究^[7]^中肾脏不良事件的发生率则可达9.9%-29%。急性肾损伤通常在ICIs治疗后数周至数月发生，其中ipilimumab治疗后多数在6周-12周，而nivolumab往往在治疗后6个月-12个月^[8]^。

## 2 病理特点

目前所有种类ICIs都有引起急性肾小管间质损伤的个案报道^[8, 9]^，尚缺乏准确的病理分型。统计已有的个案报道，绝大多数表现为急性肾小管间质性肾炎（acute tubulo-interstitial nephritis, ATIN）^[3]^。其中以淋巴细胞浸润为主，伴有不同程度的浆细胞和嗜酸性粒细胞浸润，免疫荧光大多无明显阳性染色。典型者以光镜下间质肉芽肿病变和电镜下足突消失为较特异的病理表现^[10]^。目前认为，正常组织对T细胞耐受下降，NSAIDS、PPIs及ICIs增加特异性T细胞活化以及自身抗体产生均是可能的免疫相关ATIN发病机制。此外，一些其他种类的肾小球病，如血栓性微血管病、微小病变肾病^[11]^、狼疮性肾炎^[12]^也偶见报道。

## 3 诊断及管理

ICIs治疗后急性肾小管间质性肾炎是最常见的肾脏不良事件。以肾小管间质损伤为主，单纯的肾病综合征罕见^[11]^。该类患者中血清肌酐升高发生率接近100%，部分伴有尿白细胞升高、血尿、血嗜酸性粒细胞增多以及继发高血压等^[13]^。少数患者出现低钠、低钾或低钙血症，其中低钙血症可能与继发性甲状旁腺功能减退相关^[14]^，但在更大剂量的ICIs治疗中则有待进一步证实^[6]^。因此尿常规及沉渣、24 h尿蛋白和血清肌酐是初筛最重要的指标。根据尿蛋白及肌酐水平，我们可以更合理地做出临床决策（表 1、表 2）。与其他肾小球病类似，建议尿蛋白 > 3.5 g/24 h或反复尿蛋白1 g-3.5 g/24 h作为肾活检的指征。建议PD-1抑制剂通常在用药后3个月-6个月内开始肾功能的监测，而CTLA-4抑制剂则相对更早，建议早期（< 3个月）就可开始。肿瘤患者在治疗期间发生急性肾损伤的原因较复杂，如尿常规等检查基本阴性，未除外其他继发因素时不推荐首先应用激素治疗。当监测出现血肌酐异常时，应开始肾功能异常的鉴别诊断，以区分肾前性、肾性及肾后性因素。详细询问病史，包括液体摄入、腹泻及感染、使用药物[非甾体类抗炎药（nonsteroidal antiinflammatory drugs, NSAIDs）及质子泵抑制剂（Proton pump inhibitors, PPIs）等]，完善尿常规及沉渣、肾功能及电解质、肾脏超声等辅助检查。筛查尿常规如提示白细胞或红细胞升高，应首先除外尿路感染可能。如患者此前存在长期少量血尿、蛋白尿及肾功能损伤，则首先应除外代谢疾病引起的肾损伤，如高血压、糖尿病、高尿酸血症等。根据美国国家综合癌症网络（National Comprehensive Cancer Network, NCCN）2019指南的建议，如出现符合急性肾损伤标准的情况（血清肌酐升高至基线以上1.5倍或增加0.3 mg/dL），可至少每3-7天监测一次，警惕出现严重的肾衰相关并发症（表 3）。目前血清肌酐虽不够敏感，但仍是最为经济有效的筛查方式。如出现病情明显变化或影响重要药物使用的决策，肾活检是确诊肾脏损伤的金标准。在病情发展的初期、即使肾功能损伤不严重，也应积极考虑肾活检以合理决定此后治疗方案。如肾脏病理证实ATIN，在停药及免疫抑制治疗的同时，应及时组织多学科讨论将来是否具有重启ICIs治疗的指征。

**1:** ICIs尿常规及肌酐的管理 Recommendations on management of routine urine test and serum creatine on ICIs therapy

Conditions	Work-up	ICIs management	Treatment
sCr 1-1.5×baseline UPro≥2+	Rule out other factors	Continue	Discontinue potential nephrotoxic drugs Correct prerenal factors
Leukocyturia (> 5 wbc/hpf)	Check after 1 week		
sCr 1.5-3.0×baseline UPro≥2+	Rule out other factors	Withhold therapy	Discontinue potential nephrotoxic drugs When kidney biopsy confirms ATIN:
Leukocyturia (> 5 wbc/hpf)	Consider kidney biopsy		Prednisolone 0.5 mg/kg/d-1.0 mg/kg/d or equivalent and continue until improvement to mild. Taper over 1 months
sCr > 3.0×baseline UPro≥2+	Rule out other factors	Withhold therapy	Discontinue potential nephrotoxic drugs When kidney biopsy confirms ATIN:
Leukocyturia (> 5 wbc/hpf)	Perform kidney biopsy		Prednisolone 1.0 mg/kg/d-2.0 mg/kg/d or equivalent and continue until improvement to mild.
			Taper over 1 months
ICIs: immuno-checkpoint inhibitors; sCr: serum creatinine; UPro: urinary protein.			

**2:** ICIs尿蛋白定量的管理 Recommendations on management of proteinuria on ICIs therapy

Conditions	Work-up	ICIs management	Treatment
Upro < 1 g/24 h	Rule out other factors Check sCr, urine routine and sediment,	Continue	Observe if urine sediment shows negative
Upro 1-3.5 g/24 h	Rule out other factors Check sCr, urine routine and sediment Consider kidney biopsy	When kidney biopsy confirms:Withhold therapy	Treat the diagnosed glomerular disease
Upro > 3.5 g/24 h	Perform kidney biopsy	When kidney biopsy confirms:Withhold therapy	Treat the diagnosed glomerular disease

**3:** AKI的处理（NCCN 2019V2-免疫治疗相关毒性管理） Management of AKI(NCCN 2019 V2 management of immunotherapy-related toxicities)

Conditions	Work-up	Management
Mild (Grade 1)	sCr 1-1.5×baseline or increase 0.3 mg/dL	Withhold ICIs Monitor sCr and Upro at least every 3 d-7 d
Moderate (Grade 2）	sCr 2-3×baseline	Withhold ICIs Monitor sCr and Upro at least every 3 d-7 d Nephrology consultation Rule out other factors Start prednisolone 0.5 mg/kg/d-1.0 mg/kg/d For persistent G2 > 1 week，prednisolone 1.0 mg/kg/d-2.0 mg/kg/d
Severe (Grade 3)	sCr > 3×baseline	Permanently discontinue ICIs Consider inpatient care
	or > 4 mg/dL	Nephrology consultation Consider renal biopsy Start prednisolone 1.0 mg/kg/d-2.0 mg/kg/d
Life-Threatening (Grade 4)	sCr > 6×baseline	If > G2 after 1 week of steroids Consider Immunosuppressive therapy (CTX, AZA, CsA, MMF, Infliximab)
	Or dialysis indicated	
CTX: Cyclophosphamide; AZA: Azathioprine; CsA: Cyclosporine; MMF: Mycophenolate; NCCN: National Comprehensive Cancer Network.		

## 4 治疗原则

主要包括停用药物、糖皮质激素及必要的肾脏替代治疗。除ICIs外，许多个案报道指出质子泵抑制剂和非甾体类抗炎药的应用可能导致急性肾小管间质性肾炎^[15]^，必要时应停药观察。其他可能引起肾毒性的传统药物，如氨基糖苷类和造影剂也应在急性期尽量避免使用。如肾活检确诊急性小管间质性肾炎且合并严重肾功能损伤，应考虑永久停药。在其他病因引起或肾功能损伤较轻的情况下，可根据病情需要重启治疗。激素治疗的最佳剂量和疗程尚无明确证据，可部分参照传统急性间质性肾炎的治疗方案。现有证据表明应用泼尼松0.5 mg/kg-2 mg/kg剂量以及至少1个月-2个月的减量过程，对于多数病例反应良好。如病情相对严重或迁延不愈，也可考虑适度增加激素及延长疗程。其他免疫抑制治疗如霉酚酸酯和抗肿瘤坏死因子-α（tumor necrosis factor-α, TNF-α）药物等，已逐步应用于临床，但尚需更多证据支持。此外，即使病情缓解，也同样建议在治疗后1个月-3个月继续每周监测血清肌酐水平。

## 5 预后

糖皮质激素对于ICIs所致急性肾小管间质性肾炎有较好的效果。大多数诊断明确的患者如及时干预，肾功能多数可部分甚至完全恢复，同时病情复发的情况少见^[3]^。如肾脏病理提示明确的肉芽肿病变形成，可能提示对激素的反应及肾功能恢复均不佳。此外，其他特殊类型的肾小球疾病十分罕见，预后方面仍需进一步统计。

## 6 总结

随着ICIs在肿瘤治疗的应用愈来愈广泛，T细胞过度活化带来的免疫不良事件须引起警惕。最近的数据^[7]^表明肾脏不良事件高于原先的估计。更高剂量ICIs和联合治疗中肾脏不良事件发生率更高。急性肾小管间质性肾炎是常见的ICIs肾损伤的病理类型，而其他肾小球疾病也偶有报道。具体的病理分型有待于更多的临床个例验证。尿常规及沉渣、24 h尿蛋白和血清肌酐检测是早期筛查的重要指标。初步除外其他继发因素后，如不能明确诊断，肾活检仍是确诊肾脏病变的金标准。其指征包括肾功能严重损伤以及肾病范围蛋白尿。如需作出重要诊疗决策、肾脏病理也是重要的参考。临床处理ICIs免疫相关肾脏不良事件并不简单，须根据具体情况决定个体化诊疗，往往需要肿瘤、肾脏内科甚至泌尿外科等多专科的共同协作。
